# Monitoring Digital Health Equity Across the Digital Front Door: U.S. Trends in Broadband Access, Portal Offers, and Patient IT Use in 2017–2020

**DOI:** 10.3390/healthcare14193268

**Published:** 2026-10-02

**Authors:** Rehab A. Rayan, Salman Kimiagari

**Affiliations:** 1 Economics Department, Bob Gaglardi School of Business and Economics, Thompson Rivers University, Kamloops, BC V2C 0C8, Canada; 2 Department of Epidemiology, High Institute of Public Health, Alexandria University, Alexandria 21526, Egypt; 3 Management, Information, and Supply Chain Department, Bob Gaglardi School of Business and Economics, Thompson Rivers University, Kamloops, BC V2C 0C8, Canada; skimiagari@tru.ca

**Keywords:** digital health equity, health informatics, digital divide, patient portal, broadband access, healthcare accessibility, Healthy People 2030, HINTS

## Abstract

**Highlights:**

**What are the main findings?**

- U.S. digital health indicators followed a divergent access–offer–use pathway from 2017 to 2020: broadband access declined, while portal offers and patient IT use increased.
- Socioeconomic inequalities persisted across the pathway: education gradients widened for broadband access and portal offers, while education and income gradients narrowed for downstream patient IT use.

**What are the implications of the main findings?**

- Digital health performance should not be assessed using aggregate uptake alone; access, organizational offering, and patient use should be monitored as separate but connected equity-sensitive service stages.
- Healthcare organizations, payers, and policymakers can use combined inequality indices and precision-adjusted subgroup monitoring to target connectivity screening, portal-offer workflows, digital navigation, and low-bandwidth service design.

**Abstract:**

**Background/Objectives**: Patient portals and patient-facing information technologies are now routine channels for healthcare delivery, but healthcare organizations need practical ways to monitor whether these channels are equitably accessible and used. This study examined U.S. national trends in digital health equity from 2017 to 2020 using three Healthy People/HINTS indicators representing an access–offer–use pathway: broadband access, being offered online access to medical records, and using information technology to track or communicate about care. **Methods**: We conducted a repeated cross-sectional analysis of publicly available aggregate Healthy People 2030 indicator data based on the Health Information National Trends Survey. National trends were summarized for HC/HIT-05, HC/HIT-06, and HC/HIT-07. Education- and income-related inequalities were assessed using the Slope Index of Inequality and Relative Index of Inequality. Subgroup variation was examined using precision-adjusted funnel monitoring with overdispersion adjustment. Sensitivity analyses assessed alternative overdispersion specifications, weighting approaches, and confidence-interval diagnostics. **Results**: From 2017 to 2020, broadband access declined from 55.9% to 41.5%, while portal offers increased from 61.4% to 72.0% and patient IT use increased from 80.0% to 83.3%. Socioeconomic inequalities persisted across the pathway. Education-related gradients widened for broadband access and portal offers, whereas education and income gradients narrowed for downstream patient IT use. Funnel monitoring indicated subgroup heterogeneity beyond expected sampling variation, supporting the need for equity monitoring beyond aggregate national trends. **Conclusions**: Digital health performance should not be evaluated using overall uptake alone. Monitoring access, organizational offering, and patient use as separate but connected stages can help healthcare organizations, payers, and policymakers identify where inequities occur and target connectivity screening, portal-offer workflows, digital navigation support, and low-bandwidth service design.

## 1. Introduction

Digital health is no longer an “innovation project” in healthcare; it is a routine service channel through which patients access information, transact, and communicate with care teams. As digital tools become embedded in care delivery, broadband access, portal offering, and patient IT use function as healthcare accessibility indicators rather than merely technology-adoption measures. Digital health equity frameworks emphasize that infrastructure, digital literacy, accessibility, usability, readiness, and organizational support shape whether digital tools reduce or reproduce health inequities [1,2,3,4].

For healthcare organizations, payers, and policymakers, this creates a monitoring problem: digital channel performance cannot be judged by overall uptake alone, but by whether access, organizational offering, and downstream use are equitably realized across patient segments [5].

We examine this monitoring challenge using three nationally reported Healthy People HC/HIT indicators that map onto an operational pathway: broadband connectivity (HC/HIT-05), organizational offering of online medical record access (HC/HIT-06), and patient IT use to track or communicate about care. Each stage is shaped by different management and policy actions: connectivity support for access, standardized workflows for offering, and activation, onboarding, and usability support for sustained use.

Adoption differences are not random noise. They reflect how resources and capabilities are distributed (e.g., broadband, devices, digital literacy) and how organizations implement and promote digital channels (e.g., default enrollment, encouragement scripts, language access, accessibility features). Two organizations can deploy similar portal functionality yet produce very different equity outcomes because implementation choices determine who is offered access, who activates, and who converts to meaningful use [5,6,7,8,9,10].

The 2017–2020 period is informative for management because it captures rapid channel expansion under operational pressure and allows us to observe whether offering and use rose faster than the upstream access indicator and whether inequality gradients widened or narrowed as systems scaled digital services [11]. This setting is particularly useful for distinguishing “availability” from “conversion”: systems may raise offers and certain digital behaviors even when household connectivity remains constrained.

The practical gap is not whether digital tools exist but whether they can be managed as service channels with equity as a measurable performance dimension. Most prior studies either (i) report adoption differences without a monitoring framework that separates signal from noise in small subgroups, or (ii) compare extreme groups rather than summarizing inequality across the full socioeconomic distribution. This limits decision-making on where to intervene (connectivity support vs. offering/activation workflows vs. conversion and usability) and how to hold vendors and delivery organizations accountable over time [12,13,14,15].

What remains underdeveloped in healthcare management and health informatics research is a practical way to monitor digital equity at scale: one that distinguishes persistent subgroup signals from sampling noise and summarizes socioeconomic gradients across the full distribution rather than comparing only extreme groups.

We treat equity as a managed performance outcome of the digital front door. In payer and provider settings, this means monitoring offer rates, activation-to-use conversion, and socioeconomic inequality gradients in ways that can inform outreach, digital navigation, low-bandwidth design, accessibility compliance, and vendor or contracting requirements.

The digital front door is therefore not only a technology asset but also a service-delivery pathway. Monitoring this pathway requires distinguishing three linked questions: whether patients have the infrastructure to connect, whether organizations offer online access consistently, and whether patients use digital tools to manage care. If these stages are monitored only through aggregate uptake, organizations may miss inequities that affect quality, responsiveness, and access.

### 1.1. Digital Health Equity and Healthcare Accessibility

A consistent finding across the digital divide literature is that connectivity and material resources shape who can participate in digital health at all. Lower-income and lower-education groups face affordability and capability constraints that limit reliable broadband and device access, which then constrains downstream portal and digital communication behaviors [13,16,17,18,19]. In practical terms, access is not just an individual attribute; it is a prerequisite that organizations must treat as part of service delivery infrastructure when digital channels become default. Broadband is treated here as an upstream infrastructure indicator, not as the only route to digital healthcare. Patients may use smartphones, shared devices, public internet access, or healthcare-facility kiosks to reach digital services. Nevertheless, the availability, affordability, privacy, reliability, and functionality of these alternatives vary, and the HC/HIT-05 measure does not capture all forms of effective digital access [1,3,4,18].

Digital exclusion reflects broader social determinants of health and can be compounded for people with disability and chronic conditions, who may benefit from digital tools yet face greater access and usability barriers. Without deliberate equity-oriented implementation, digital channel expansion can widen rather than reduce disparities [8,9,12,20,21,22,23].

Even when access exists, use depends on whether organizations systematically offer online access and make activation easy. Organizations can improve offering through standardized enrollment scripts, default activation prompts, staff encouragement, accessible language options, and low-bandwidth pathways. Evidence also suggests that older adults and other underserved groups may be disproportionately affected when offering/activation processes assume high digital literacy or lack tailored supports [8,9].

Existing studies have identified important individual, organizational, and structural determinants of digital health inequity, and recent frameworks provide guidance for considering equity across the digital healthcare lifecycle [1,2,3,4]. However, the integration of these considerations into a repeatable monitoring approach that simultaneously summarizes socioeconomic inequality across the full distribution and distinguishes subgroup signals from sampling variability remains underdeveloped. This limits the ability of healthcare organizations and payers to identify where inequities emerge across access, offering, and use and to monitor those patterns consistently over time.

### 1.2. The Digital Front Door as an Access–Offer–Use Pathway

We conceptualize the digital front door as a service pathway with three stages. HC/HIT-05 represents an upstream connectivity indicator (broadband), HC/HIT-06 represents organizational offering (being offered online medical record access), and HC/HIT-07 represents downstream digital behavior (using IT to track or communicate about care). Socioeconomic status, measured by education and income, is the primary inequality domain because it captures gradients across the full distribution and supports annual monitoring. Other subgroup domains, including age, sex, race/ethnicity, geography, marital status, and disability, are monitored descriptively and through funnel plots to support operational oversight [24,25,26].

### 1.3. Study Aim and Contribution

The novelty of this study lies in integrating three nationally reported digital health indicators into a single access–offer–use monitoring pathway and combining distribution-wide socioeconomic inequality indices with precision-adjusted subgroup monitoring. The individual indicators and analytical methods are established; their integration provides a practical framework that distinguishes structural access, organizational offering, and downstream use while separating systematic subgroup signals from sampling variability. The framework also translates national indicator evidence into practical priorities for healthcare organizations, payers, and policymakers, including connectivity screening, standardized portal-offer workflows, digital navigation support, and low-bandwidth service design.

We organize prior work around the service pathway used in our analysis—access → offering → use—because it maps directly to healthcare management and health informatics decisions. “Access” reflects structural prerequisites such as connectivity and devices; “offering” reflects organizational implementation, including workflows and vendor configuration; and “use” reflects activation, onboarding, usability, and sustained engagement.

Guided by this pathway and monitoring framing, we investigate:

1. RQ1: How did national performance across the digital health service pathway, including broadband access, portal offering, and patient IT use, change from 2017 to 2020?
2. RQ2: How did education- and income-related inequalities in each pathway stage change over time?
3. RQ3: How can precision-adjusted subgroup monitoring support healthcare organizations, payers, and policymakers in identifying equity signals beyond aggregate uptake?

## 2. Materials and Methods

### 2.1. Study Design and Data Source

We conducted a repeated cross-sectional analysis of nationally reported Healthy People 2030 Health Communication/Health Information Technology indicators for 2017–2020. The indicators are based on the Health Information National Trends Survey (HINTS), which provides national and subgroup point estimates with standard errors for Healthy People 2030 objectives [27].

HINTS is a repeated cross-sectional survey designed to produce nationally representative estimates for noninstitutionalized U.S. adults. During the study period, it used address-based sampling and predominantly mailed, self-administered questionnaires, with full-sample weights used to account for selection probabilities and nonresponse, and replicate weights used for variance estimation. Nevertheless, the estimates remain subject to self-report error, nonresponse bias, and underrepresentation of people outside the household sampling frame or those facing barriers to survey participation [27].

We examined three indicators representing a digital health service pathway: broadband access (HC/HIT-05), being offered online medical record access (HC/HIT-06), and using information technology to track or communicate about care (HC/HIT-07). The analysis was designed for digital health equity monitoring rather than causal inference. Detailed HINTS cycle information, field windows, and technical data notes are provided in Supplementary File S1. Their pathway stages, management interpretations, and principal metrics are summarized in Table 1.

The HINTS cycles included 3191 respondents in 2017, 3504 in 2018, 5247 in 2019, and 3865 in 2020. These are cycle-level unweighted survey sample sizes and should not be interpreted as indicator-specific denominators. Cycle details, data availability, national estimates, and standard errors are provided in Supplementary Tables S1 and S2.

Individual adults were the underlying HINTS respondents; however, the observational units in the present aggregate analysis were published indicator-year estimates for national totals and published subgroup-by-year estimates for inequality and funnel monitoring.

Published aggregate estimates were selected because the study aimed to evaluate a monitoring approach that could be implemented using routinely reported Healthy People indicators rather than requiring restricted or individual-level survey data. This choice improves alignment with public reporting and reproducibility but limits covariate adjustment, interaction analysis, and causal inference.

The final observation year, 2020, overlapped with the first year of the COVID-19 pandemic. The period should therefore be interpreted as spanning both pre-pandemic years and an early-pandemic observation rather than as a stable pre-pandemic time series.

### 2.2. Digital Health Service Pathway Indicators

- HC/HIT-05: Increase the proportion of adults with broadband internet. Topic-area placement in Healthy People 2030 reflects its enabling role for digital health [26].
- HC/HIT-06: Increase the proportion of adults offered online access to their medical records by a healthcare provider or insurer [24].
- HC/HIT-07: Increase the proportion of adults who use IT to track healthcare data or communicate with providers [25].

For each indicator, the outcome metric is the percentage (0–100) for the national total and subgroups by year; totals serve as the funnel centers in precision-adjusted comparisons.

### 2.3. Socioeconomic Domains and Subgroup Monitoring

Education and income were the primary ordered socioeconomic stratifiers, not outcome variables. They were selected because their categories can be meaningfully ranked from lower to higher socioeconomic advantage, which is required for distribution-wide SII and RII estimation. Other characteristics, including age, sex, race/ethnicity, marital status, geography, and disability, were not treated as a single socioeconomic hierarchy and were therefore assessed descriptively and through subgroup funnel monitoring.

### 2.4. Socioeconomic Inequality Measures

For each indicator-year, education and income categories were ordered from least to most advantaged and assigned relative rank scores representing their positions across the socioeconomic distribution. We regressed the subgroup outcome percentage on the rank score. The Slope Index of Inequality (SII) was defined as the estimated difference in the predicted outcome between the highest and lowest ends of the socioeconomic distribution and is reported in percentage points. The Relative Index of Inequality (RII) was calculated as the ratio of the predicted outcome at the highest rank to that at the lowest rank. Positive SII values and RII values greater than 1 indicate higher outcomes among more advantaged groups [28,29]. Equal-category weighting was used in the primary analysis, with population-share weighting examined in sensitivity analysis. Additional formulas and interpretation are provided in Supplementary File S1, and the indicator-specific SII/RII trajectories are shown in Supplementary Figures S1–S6.

### 2.5. Precision-Adjusted Funnel Monitoring

Precision-adjusted funnel plots were centered on the national estimate for each indicator-year. Subgroup estimates were plotted against their variance-equivalent effective sample sizes, approximated from each published proportion and standard error as $n_{eff}=p\left(1-p\right)/SE^{2}$. When estimates and standard errors were reported as percentages, both were converted to proportions before the effective sample size was calculated. Expected limits were calculated around the national estimate using subgroup-specific uncertainty. Because observed subgroup dispersion exceeded simple binomial expectations, the limits were multiplied by the square root of an estimated overdispersion factor. A 10% Winsor specification was used in the primary analysis to reduce the influence of extreme standardized residuals, and 5% and 20% specifications were examined in sensitivity analyses. Subgroups outside the adjusted 95% and 99.8% limits were treated as monitoring signals rather than causal outliers [30]. Technical formulas and selected sensitivity results are provided in Supplementary File S1.

### 2.6. Sensitivity Analyses

Sensitivity analyses assessed whether findings were robust to alternative overdispersion specifications (5%, 10%, and 20% Winsor levels), alternative weighting of SII/RII models, and confidence-interval methods for proportions near 0% or 100%. Selected sensitivity outputs are reported in Supplementary File S1 [31,32].

### 2.7. Ethics and Data Availability

All analyses used publicly available aggregate Healthy People objective data based on HINTS indicators; no individual-level data were analyzed, and no IRB review was required. Data for HC/HIT-05, HC/HIT-06, and HC/HIT-07 are publicly available from Healthy People 2030 objective pages [24,25,26]. Derived tables, figures, and technical details are provided in Supplementary File S1.

## 3. Results

### 3.1. National Pathway Trends

National performance diverged across the digital health service pathway (Table 2). Broadband access declined by 14.4 percentage points, from 55.9% in 2017 to 41.5% in 2020. Over the same period, portal offers increased by 10.6 percentage points, from 61.4% to 72.0%, and patient IT use increased by 3.3 percentage points, from 80.0% to 83.3% [33].

Figure 1 displays these trends graphically, highlighting the divergence between broadband access and health IT engagement. Broadband access (HC/HIT-05) declined between the 2017 baseline and 2020, whereas being offered online access to medical records (HC/HIT-06) and using health IT to manage or communicate about care (HC/HIT-07) increased through 2020, with persistent socioeconomic disparities across education and income subgroups [33].

### 3.2. Socioeconomic Inequality Gradients

Education and income-related gradients were evident across the indicators throughout 2017–2020: higher education and income groups consistently reported greater broadband access and higher rates of being offered and using digital health tools. These disparities have been persistent and sizable in the HINTS series, with underserved groups (lower SES, older adults, racial/ethnic minorities, and persons with disabilities) less likely to be offered or to use portals and other health IT.

For the ordered socioeconomic domains of education and income, SII and RII estimates showed pro-advantaged gradients in each year: outcomes were higher at the more advantaged end of the socioeconomic distribution than at the less advantaged end. The magnitude of the gradient differed by indicator and socioeconomic domain.

Across 2017–2020, all estimated gradients remained pro-advantaged. Their magnitude varied by indicator and socioeconomic domain. Population-share weighting did not change the qualitative direction of the findings. Table 3 presents the 2017 and 2020 SII and RII estimates; 2017–2020 change summaries are provided in Supplementary Table S3, and intermediate-year trajectories are shown in Supplementary Figures S1–S6.

These patterns indicate that inequalities widened at earlier stages of the pathway (access and offering) while narrowing at the downstream use stage. Inequality patterns differed across stages, with the largest absolute gradients observed in IT use (HC/HIT-07) despite overall high uptake, indicating that widespread adoption does not eliminate socioeconomic differences in use intensity or type.

Education-related inequality widened for HC/HIT 05 and HC/HIT 06 but narrowed for HC/HIT 07. For HC/HIT 05, SII increased from 27.4 pp (2017) to 31.5 pp (2020) and RII from 1.68 to 2.19. For HC/HIT 06, SII rose sharply from 24.9 pp to 46.2 pp, while RII increased from 1.55 to 1.90. Conversely, HC/HIT 07 saw SII decline from 52.3 pp to 39.8 pp and RII from 1.98 to 1.60.

Income gradients were stable for HC/HIT 05 and HC/HIT 06 but narrowed for HC/HIT 07. For HC/HIT 07, SII dropped from 37.0 pp to 18.4 pp and RII from 1.59 to 1.29.

### 3.3. Precision-Adjusted Subgroup Monitoring

Precision-adjusted monitoring identified subgroup estimates outside the overdispersion-adjusted control limits, indicating variation greater than expected from the reported subgroup uncertainty alone. Below-center signals were concentrated among lower socioeconomic groups for broadband access and portal offering. The available 2020 funnel plots in Supplementary Figures S7–S9 illustrate these patterns, and selected outlier counts are reported in Supplementary Table S5. Adjustment for overdispersion reduced the number of flagged estimates, but below-center signals remained evident. These findings identify subgroups for further monitoring; they do not establish causal effects or rank subgroup performance [30].

### 3.4. Sensitivity Checks

Sensitivity checks supported the main findings. SII/RII estimates were directionally consistent under alternative weighting, and funnel-based subgroup signals were stable across 5%, 10%, and 20% Winsor specifications. Confidence-interval diagnostics for extreme subgroup proportions did not alter substantive conclusions. Selected sensitivity results are reported in Supplementary Tables S4 and S5 [31,32].

## 4. Discussion

### 4.1. Principal Findings

Using nationally reported Healthy People HC/HIT indicators (2017–2020), the study found a divergence across the digital health pathway: broadband access (HC/HIT 05) declined from the 2017 baseline to 2020, whereas being offered online medical record access (HC/HIT 06) and using IT to track or communicate about care (HC/HIT 07) rose modestly over the same period. Education and income-based inequalities were persistent: education gradients widened for HC/HIT 05 and HC/HIT 06, while income and education gradients narrowed for HC/HIT 07. This pattern is consistent with a divergent service pathway in which organizational adoption and encouragement increased portal offers and use, even as household connectivity constraints limited progress on the upstream access indicator. These results align with Healthy People 2030 equity performance monitoring that classifies HC/HIT 05 as “getting worse” (55.9% in 2017 to 41.5% in 2020) [26].

### 4.2. Implications for Healthcare Organizations and Payers

For healthcare organizations, payers, and policymakers, the findings suggest that digital access, organizational offering, and patient use should be monitored as distinct but connected service stages. Improving portal availability alone may not address inequity if patients lack reliable broadband, devices, language support, or digital navigation assistance. Health systems and payers can respond by screening for connectivity barriers, standardizing portal-offer workflows, monitoring activation-to-use conversion, and using subgroup equity signals to target digital navigation and low-bandwidth service design.

The divergence between access and use suggests that healthcare organizations, payers, and policymakers should not treat portal availability as evidence of equitable digital access. Organizational policies, EHR diffusion, and staff encouragement may increase offers and use, but household-level barriers such as broadband affordability, device availability, language access, and digital literacy can still constrain equitable participation. Management dashboards should therefore separate access, offering, activation, and use rather than reporting a single digital uptake measure [33,34,35].

### 4.3. Interpretation of Pathway Dynamics

The access–offer–use pathway operationalizes digital health equity as a measurable healthcare service pathway. This framing complements broader digital health equity frameworks that identify digital access, usability, literacy, readiness, and organizational support as determinants of equitable digital care. In this study, broadband access represents infrastructure readiness, portal offering represents organizational implementation, and patient IT use reflects downstream engagement. Monitoring these stages separately can help healthcare organizations avoid treating digital uptake as a single performance indicator when the underlying barriers differ across the pathway [1,2,3,4].

The narrowing of inequalities in downstream IT use is notable. This pattern may indicate that once access barriers are overcome, either through smartphones, shared devices, or assisted access, patients across socioeconomic groups can engage with digital tools [34,36]. It may also reflect targeted encouragement by providers or the diffusion of simpler digital behaviors compared to more resource-dependent access measures.

HINTS and other national surveillance systems, including the American Community Survey, use different questions, sampling approaches, populations, and units of analysis to measure digital access [37,38]. These differences reinforce the need to interpret the HC/HIT-05 trend as a HINTS/Healthy People indicator rather than as a comprehensive estimate of all fixed, mobile, shared, or public internet access.

The decline in HC/HIT-05 should be interpreted cautiously. Healthy People 2030 classifies this indicator as “getting worse,” based on a decline from 55.9% in 2017 to 41.5% in 2020 [26]. However, HC/HIT-05 is a survey-specific measure and is not equivalent to every national measure of household internet subscription. The observed pattern may reflect affordability or reliability constraints, substitution toward mobile-only or shared access, changes in household circumstances, survey measurement, or the exceptional conditions of 2020. Accordingly, the finding should not be interpreted as evidence that all forms of U.S. internet connectivity declined during this period.

Taken together, the findings suggest that different mechanisms operate at each stage of the digital front door. Access remains structurally constrained, offering is organizationally driven, and use is shaped by both capability and support. This reinforces the value of monitoring the pathway as a sequence rather than relying on single indicators.

### 4.4. International Relevance

Although the analysis uses U.S. national indicators, the management problem is relevant to health systems in other high-income countries where patient portals, telehealth, and digital communication tools are increasingly embedded in routine care. The pathway logic, access, organizational offering, and patient use can be adapted to different national data systems. The specific indicator values will vary by country, but the management risk is general: aggregate digital progress can coexist with widening socioeconomic gaps if organizations do not monitor equity across the full service pathway. This concern extends beyond the United States. The WHO Regional Office for Europe has reported uneven uptake of digital services and substantial variation in national policies to improve digital health literacy, demonstrating that digital transformation can leave some population groups behind [39].

### 4.5. Practical Priorities for Healthcare Organizations, Payers, and Policymakers

Three priorities follow from the findings.

First, health systems should treat broadband access as part of care infrastructure by incorporating connectivity screening into routine workflows, particularly for patients enrolled in digital communication or remote monitoring programs. This aligns with evidence that digital literacy, connectivity, and material access shape participation in digital health services and with digital healthcare equity frameworks that emphasize infrastructure and readiness as equity-relevant factors [1,4,16,18].

Healthcare organizations cannot independently expand broadband infrastructure, but they can document connectivity barriers, preserve non-digital and low-bandwidth alternatives, and share stratified access indicators with public agencies. Policymakers can use these data to target affordability programs, infrastructure investment, digital-literacy initiatives, and accountability requirements in areas where healthcare digitization is advancing faster than effective access.

Second, organizations should standardize portal-offer and activation workflows. Variation in offering practices can reinforce inequalities if access depends on individual provider behavior or patient initiative. Embedding default enrollment processes and consistent encouragement scripts can improve offering rates across patient groups, consistent with evidence on provider encouragement and portal uptake [33,34].

Third, equity monitoring should integrate aggregate performance measures with socioeconomic gradients and subgroup signals. Dashboards that separately track access, offering, and use can help identify where breakdowns occur in the pathway. This enables more targeted interventions, such as digital navigation support, multilingual outreach, and low-bandwidth service options for patients facing structural or capability barriers. Combining inequality indices with funnel monitoring can support both distribution-wide equity assessment and subgroup signal detection [28,29,30].

The relevance of this monitoring approach has increased as healthcare access expands beyond portals to online appointment scheduling, conversational chatbots, AI-supported information services, and other digitally mediated entry points. These tools can improve convenience, but they may also reproduce inequities when access, language, usability, data quality, and algorithmic performance are not assessed across population groups [40].

### 4.6. Strengths and Limitations

A strength of this study is that it uses nationally reported indicators with standard errors and organizes them into a management-relevant service pathway linking access, organizational offering, and patient use. The analysis also combines absolute and relative socioeconomic inequality measures with precision-adjusted subgroup monitoring, allowing equity to be assessed both across the socioeconomic distribution and within smaller subgroups.

The study also has limitations. First, analyses used published aggregate estimates rather than HINTS microdata, so subgroup analyses were limited to reported categories and standard errors. Second, effective sample sizes derived from standard errors were used only as variance-equivalent diagnostics for funnel monitoring and confidence-interval sensitivity checks; they should not be interpreted as actual survey denominators. Third, the 2017–2020 period provides only four annual points for broadband and three for portal offers and IT use, limiting formal time-series inference. Finally, the study is descriptive and intended for monitoring rather than causal attribution. Taken together, these limitations mean that the findings should be interpreted as a descriptive equity-monitoring framework rather than as evidence of causal effects of specific policies or organizational interventions. HC/HIT-05 captures the reported broadband indicator but does not capture all alternative routes to digital care, including mobile-only access, shared devices, public access points, or facility-based kiosks.

In addition, the 2020 observation coincided with the first year of the COVID-19 pandemic, which accelerated digital service adoption while potentially affecting survey participation, household connectivity needs, and healthcare-seeking behavior. The study design cannot isolate the effects of the pandemic from other temporal or measurement-related influences.

## 5. Conclusions

From 2017 to 2020, U.S. digital health indicators showed a divergence across the digital front-door pathway: broadband access declined, while portal offers and patient IT use increased. Socioeconomic inequalities persisted, with widening education gradients for broadband access and portal offers and narrowing gradients for downstream IT use. These findings show why digital health performance should not be assessed using aggregate uptake alone. Healthcare organizations, payers, and policymakers should distinguish access, offering, and use and should pair aggregate performance measures with socioeconomic inequality gradients and subgroup signals. Managing the digital front door as an equity-sensitive healthcare service pathway can support targeted workflow redesign, digital navigation, low-bandwidth service models, and more inclusive digital health implementation.

## Figures and Tables

**Figure 1:**
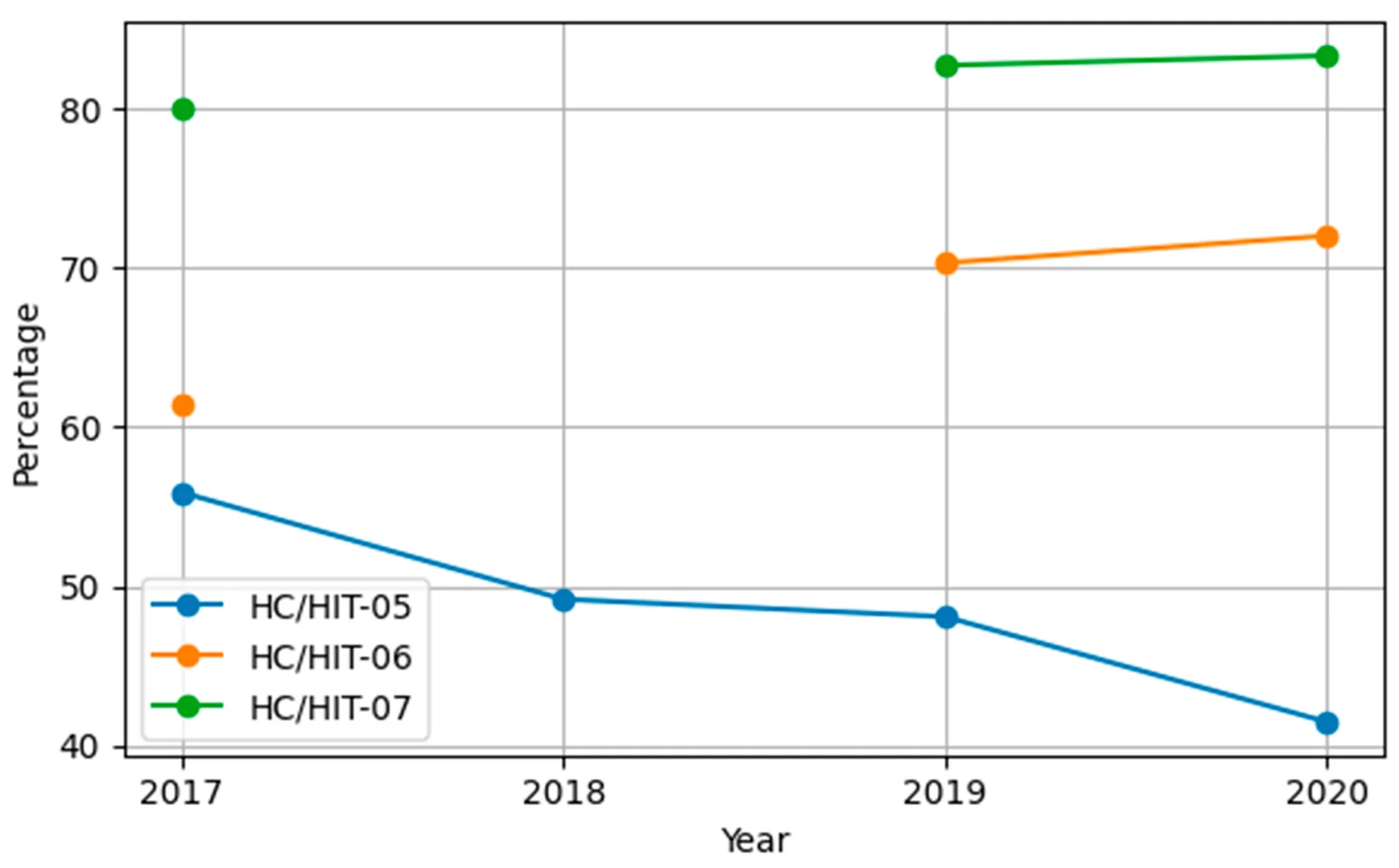
National totals for HC/HIT-05, HC/HIT-06, and HC/HIT-07, 2017–2020. Error bars represent dataset-provided 95% confidence intervals. HC/HIT-05 declined nationally from 55.9% to 41.5%; HC/HIT-06 increased from 61.4% to 72.0%; and HC/HIT-07 increased from 80.0% to 83.3%.

**Table 1:** Digital health service pathway indicators and management interpretation.

Stage	Healthy People Indicator	Management Interpretation	Main Metric
Access	HC/HIT-05	Upstream connectivity indicator	% adults with broadband
Offering	HC/HIT-06	Organizational offer of online record access	% offered access
Use	HC/HIT-07	Patient digital engagement	% using IT for care

Data management and preparation of analytical outputs were conducted using Microsoft Excel for Microsoft 365 (Microsoft Corporation, Redmond, WA, USA), and statistical analyses were conducted using IBM SPSS Statistics, version 27 (IBM Corp., Armonk, NY, USA).

**Table 2:** National totals and changes in digital health service pathway indicators: USA, 2017–2020.

Indicator	2017, % (95% CI)	2020, % (95% CI)	Change, pp
Broadband access	55.9 (52.5–59.3)	41.5 (38.2–44.8)	−14.4
Portal offers	61.4 (58.6–64.2)	72.0 (69.5–74.5)	+10.6
Patient IT use	80.0 (78.1–81.9)	83.3 (81.5–85.1)	+3.3

Note: Approximate 95% confidence intervals were calculated as the published estimate ± 1.96 times the published standard error. CI, confidence interval; pp, percentage points.

**Table 3:** Socioeconomic inequality gradients by digital health service pathway stage: 2017 and 2020.

Indicator	SES Domain	2017 SII (pp)	2017 RII	2020 SII (pp)	2020 RII
HC/HIT-05 (Broadband)	Education	27.4	1.68	31.5	2.19
	Income	27.1	1.62	26.2	2.20
HC/HIT-06 (Offered portal)	Education	24.9	1.55	46.2	1.90
	Income	29.9	1.67	28.8	1.52
HC/HIT-07 (Used IT)	Education	52.3	1.98	39.8	1.60
	Income	37.0	1.59	18.4	1.29

Note. SII values are absolute percentage-point gradients across the socioeconomic distribution; RII values are relative gradients. Positive SII and RII > 1 indicate higher outcomes among more advantaged groups.

## Data Availability

All analytic inputs are publicly available from the Healthy People 2030 objective pages for HC/HIT-05, HC/HIT-06, and HC/HIT-07 and from HINTS documentation. The Healthy People 2030 Health Information Technology objective pages are available at https://odphp.health.gov/healthypeople/objectives-and-data/browse-objectives/health-it (accessed on 9 September 2026). Derived tables, figures, and technical details are provided in Supplementary File S1.

## Supplementary Materials

The following supporting information can be downloaded at https://www.mdpi.com/article/10.3390/healthcare14193268/s1: File S1: Technical methods, additional results, and sensitivity analyses; Table S1: HINTS cycles, field windows, sample sizes, and indicator coverage; Table S2: National totals and standard errors by indicator and year; Table S3: Change in national totals and socioeconomic inequality gradients, 2017–2020; Table S4: Selected overdispersion sensitivity estimates across Winsor specifications; Table S5: Selected funnel outlier counts under the primary 10% Winsor specification; Figure S1: HC/HIT-05 Slope Index of Inequality by year, equal-category weights; Figure S2: HC/HIT-05 Relative Index of Inequality by year, equal-category weights; Figure S3: HC/HIT-06 Slope Index of Inequality by year, equal-category weights; Figure S4: HC/HIT-06 Relative Index of Inequality by year, equal-category weights; Figure S5: HC/HIT-07 Slope Index of Inequality by year, equal-category weights; Figure S6: HC/HIT-07 Relative Index of Inequality by year, equal-category weights; Figure S7: Funnel plot for HC/HIT-05, 2020; Figure S8: Funnel plot for HC/HIT-06, 2020; Figure S9: Funnel plot for HC/HIT-07, 2020.

## Abbreviations

The following abbreviations are used in this manuscript:

HC/HIT	Health Communication and Health Information Technology
HINTS	Health Information National Trends Survey
SII	Slope Index of Inequality
RII	Relative Index of Inequality
SES	Socioeconomic status
SE	Standard error
IT	Information technology
ODPHP	Office of Disease Prevention and Health Promotion
EHR	Electronic health record
