# Supplementary material for: Monitoring Digital Health Equity Across the Digital Front Door: U.S. Trends in Broadband Access, Portal Offers, and Patient IT Use in 2017–2020
Source: Healthcare (Basel). 2026 Oct 2;14(19):3268. doi: 10.3390/healthcare14193268 (PMC13665087; doi:10.3390/healthcare14193268)
Supplement: Supplementary file 1 [file healthcare-14-03268-s001.zip › healthcare-4450327-supplementary.pdf]

Supplementary File S1

Technical methods, additional results, and sensitivity analyses

S1. Data sources and indicator definitions

This study used publicly available Healthy People 2030 Health Communication and Health Information Technology (HC/HIT) objective data for three indicators related to digital health service access, offering, and use. The underlying data source for these indicators is the Health Information National Trends Survey (HINTS), which provides nationally reported point estimates and standard errors for national totals and demographic subgroups. The analysis covered available annual estimates from 2017 to 2020, corresponding to the early Healthy People 2030 baseline and follow-up period used in the main manuscript.

The three indicators were treated as a digital health service pathway:

- **HC/HIT-05:** proportion of adults with broadband internet access, representing the upstream access prerequisite for digital health participation.
- **HC/HIT-06:** proportion of adults offered online access to their medical records by a health care provider or insurer, representing organizational offering of digital access.
- **HC/HIT-07:** proportion of adults who use information technology to track health care data or communicate with providers, representing downstream patient digital engagement.

Published estimates were used as reported. The analysis did not re-estimate HINTS microdata. Point estimates and standard errors were extracted from public Healthy People objective data tables and used for descriptive trend analysis, inequality estimation, funnel plot monitoring, and sensitivity checks.

Table S1. HINTS cycles, field windows, sample sizes, and indicator coverage

Year	HINTS cycle	Field window	Unweighted n	HC/HIT-05 adults with broadband (%)	HC/HIT-06 adults offered access (%)	HC/HIT-07 adults using IT (%)
**2017**	H5-C1	Jan–May 2017	3,191	55.9	61.4	80.0
**2018**	H5-C2	Jan–May 2018	3,504	49.2	—	—
**2019**	H5-C3	Jan–Apr/May 2019	5,247	48.1	70.3	82.7
**2020**	H5-C4	Feb–Jun 2020	3,865	41.5	72.0	83.3

Note. National estimates were extracted from Healthy People 2030 objective data linked to HINTS. Dashes indicate that the indicator-year was not available in the extracted series.

The main manuscript reports national changes from 2017 to 2020. Table S1 provides additional detail on the HINTS cycles, field windows, unweighted sample sizes, and indicator availability by year. Table S2 provides national totals and standard errors for each available indicator-year. These supplementary tables support interpretation of the national trends and clarify that HC/HIT-06 and HC/HIT-07 were not available for 2018 in the extracted Healthy People/HINTS series.

Table S2. National totals and standard errors by indicator and year

**Indicator**	**2017**	**2018**	**2019**	**2020**
**HC/HIT-05: Broadband access**	55.9 (1.75)	49.2 (1.60)	48.1 (1.28)	41.5 (1.70)
**HC/HIT-06: Offered portal access**	61.4 (1.44)	—	70.3 (1.39)	72.0 (1.29)
**HC/HIT-07: Used IT for care**	80.0 (0.97)	—	82.7 (0.78)	83.3 (0.91)

Note**.** Values are percentages with standard errors in parentheses. Dashes indicate unavailable indicator-years in the extracted Healthy People/HINTS series.

S2. Socioeconomic domains and subgroup definitions

Education and income were treated as the primary ordered socioeconomic domains because they capture graded differences across the socioeconomic distribution and allow estimation of summary inequality measures. Education and income were used to estimate the Slope Index of Inequality (SII) and Relative Index of Inequality (RII).

The education categories were treated as ordered from least to most advantaged:

1. Less than high school
2. High school graduate
3. Some college
4. Bachelor’s degree or higher

The income categories were treated as ordered from least to most advantaged:

1. <$20,000
2. $20,000–<$35,000
3. $35,000–<$50,000
4. $50,000–<$75,000
5. ≥$75,000

Other subgroup domains were used for descriptive subgroup monitoring and funnel plot visualization, but not for SII/RII estimation because they are not naturally ordered socioeconomic scales. These domains included age, sex, race/ethnicity, geography, marital status, and disability status, depending on availability in the Healthy People objective data.

For funnel monitoring, subgroup estimates were compared with the corresponding national total for each indicator-year. This approach allowed subgroup variation to be assessed while maintaining consistency with the publicly reported Healthy People estimates used in the main analysis.

S3. Inequality measures: Slope Index of Inequality and Relative Index of Inequality

The main manuscript reports education- and income-related inequality using two complementary measures: the Slope Index of Inequality (SII) and the Relative Index of Inequality (RII). SII summarizes absolute inequality across the socioeconomic distribution in percentage points, whereas RII summarizes relative inequality as a ratio. Positive SII values and RII values greater than 1 indicate higher outcomes among more advantaged socioeconomic groups.

For each indicator-year, ordered socioeconomic categories were assigned a relative rank score. These rank scores represent each category's position across the cumulative socioeconomic distribution and support regression-based estimation of inequality across the full distribution rather than comparing only the lowest and highest categories.

The SII was estimated using a regression model of the subgroup outcome on the socioeconomic rank score:

$Y_i = \beta 0 + \beta 1 R_i + \varepsilon_i$

Where:

1. Y_i = outcome percentage for socioeconomic category i
2. R_i = socioeconomic rank score for category i
3. $\beta 1$ = Slope Index of Inequality

In this framework, the SII represents the estimated absolute percentage-point difference between the most and least advantaged ends of the socioeconomic distribution. For example, an SII of 30 percentage points indicates that the outcome is estimated to be 30 percentage points higher at the most advantaged end of the distribution than at the least advantaged end.

The RII was estimated as the ratio of predicted outcomes at the most advantaged versus least advantaged ends of the socioeconomic distribution:

RII = predicted outcome at highest socioeconomic rank / predicted outcome at lowest socioeconomic rank

An RII greater than 1 indicates pro-advantaged inequality. For example, an RII of 2.0 indicates that the outcome is estimated to be twice as high at the most advantaged end of the socioeconomic distribution as at the least advantaged end.

SII and RII were estimated separately for education and income for each available indicator-year. The main manuscript reports selected 2017 and 2020 estimates. Supplementary Table S3 provides 2017–2020 change summaries, and Supplementary Figures S1–S6 show available SII/RII trend plots.

S4. Effective sample-size approximation

Because the analysis used published aggregate estimates and standard errors rather than HINTS microdata, true subgroup denominators were not always available in the analytic dataset. To

support funnel plot construction and confidence-interval diagnostics, variance-equivalent effective sample sizes were approximated from published proportions and standard errors.

The effective sample size was approximated using the binomial variance relationship:

$n\_eff = p(1 - p) / SE^2$

where:

1. p = published proportion
2. SE = published standard error
3. n_eff = variance-equivalent effective sample size

When Healthy People estimates were reported as percentages, values were first converted to proportions before applying the formula. The resulting effective sample sizes were used only for variance approximation in funnel monitoring and confidence-interval sensitivity checks.

These effective sample sizes should not be interpreted as actual unweighted HINTS sample sizes, actual survey denominators, or complex-survey denominators. They are diagnostic quantities derived from reported standard errors and therefore reflect both sampling variability and design features embedded in the published standard errors.

This limitation is also noted in the main manuscript, where the study is explicitly described as a descriptive monitoring analysis based on published aggregate estimates rather than a re-analysis of individual-level HINTS microdata.

S5. Funnel plot construction and overdispersion adjustment

Funnel plots were used to support precision-adjusted subgroup monitoring. For each indicator-year, the national estimate served as the center of the funnel plot, and subgroup estimates were plotted against their corresponding precision. This approach allowed subgroup estimates to be evaluated relative to expected sampling variability, rather than relying only on crude differences from the national total.

The general form of the control limits was:

$p0 \pm z \times SE\_g$

where:

1. p0 = national estimate for the indicator-year
2. SE_g = standard error for subgroup g
3. z = standard normal

Because observed subgroup variation exceeded simple binomial expectations, control limits were adjusted for overdispersion. The overdispersion-adjusted limits were calculated as:

$p0 \pm z \times \sqrt{\varphi} \times SE\_g$

where:

1. φ = estimated overdispersion factor

The primary analysis used a 10% Winsor specification to reduce the influence of extreme subgroup residuals when estimating the overdispersion factor. Sensitivity analyses used 5% and 20% Winsor specifications to assess whether subgroup signals were stable across alternative overdispersion assumptions.

Subgroups outside overdispersion-adjusted control limits were interpreted as potential equity signals for monitoring purposes. They were not interpreted as causal outliers or evidence that a subgroup's estimate was caused by a specific policy, organization, or intervention. This interpretation is consistent with the descriptive monitoring purpose of the analysis.

S6. Socioeconomic inequality results

The main manuscript reports selected 2017 and 2020 SII/RII estimates. Table S3 provides the corresponding 2017→2020 changes in national totals and socioeconomic inequality gradients. Positive SII values indicate higher estimates among more advantaged socioeconomic groups, and RII values greater than 1 indicate pro-advantaged relative inequality.

Table S3. Change in national totals and socioeconomic inequality gradients, 2017–2020

Indicator	Δ Total (pp)	Δ SII Education (pp)	Δ RII Education	Δ SII Income (pp)	Δ RII Income
**HC/HIT-05**	–14.4	+4.1	+0.51	–0.9	+0.58
**HC/HIT-06**	+10.6	+21.3	+0.35	–1.1	–0.15
**HC/HIT-07**	+3.3	–12.4	–0.37	–18.7	–0.30

Note**.** Changes are calculated as 2020 minus 2017. SII values are expressed in percentage points. RII values are ratios.

Supplementary Figures S1–S6 display available SII and RII trend plots by indicator and socioeconomic domain.

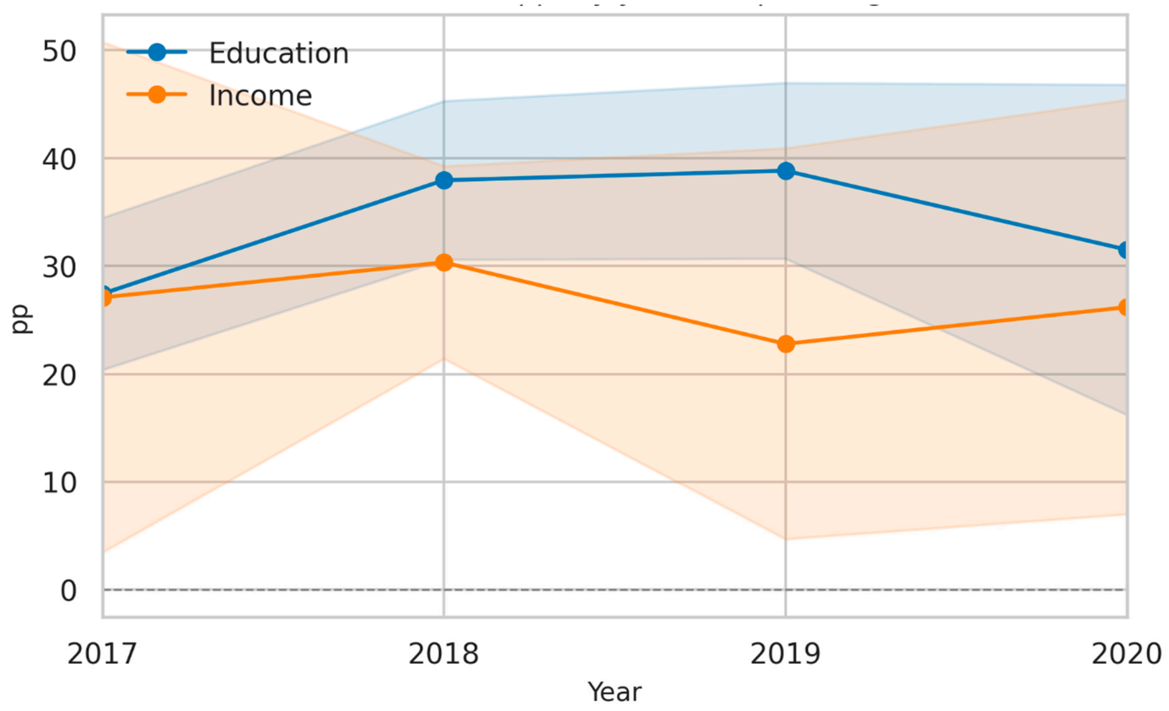

Figure S1. HC/HIT-05: Slope Index of Inequality by year, equal-category weights.

Note. SII values are expressed in percentage points. The horizontal reference line indicates no absolute socioeconomic gradient. Bands represent 95% confidence intervals where available.

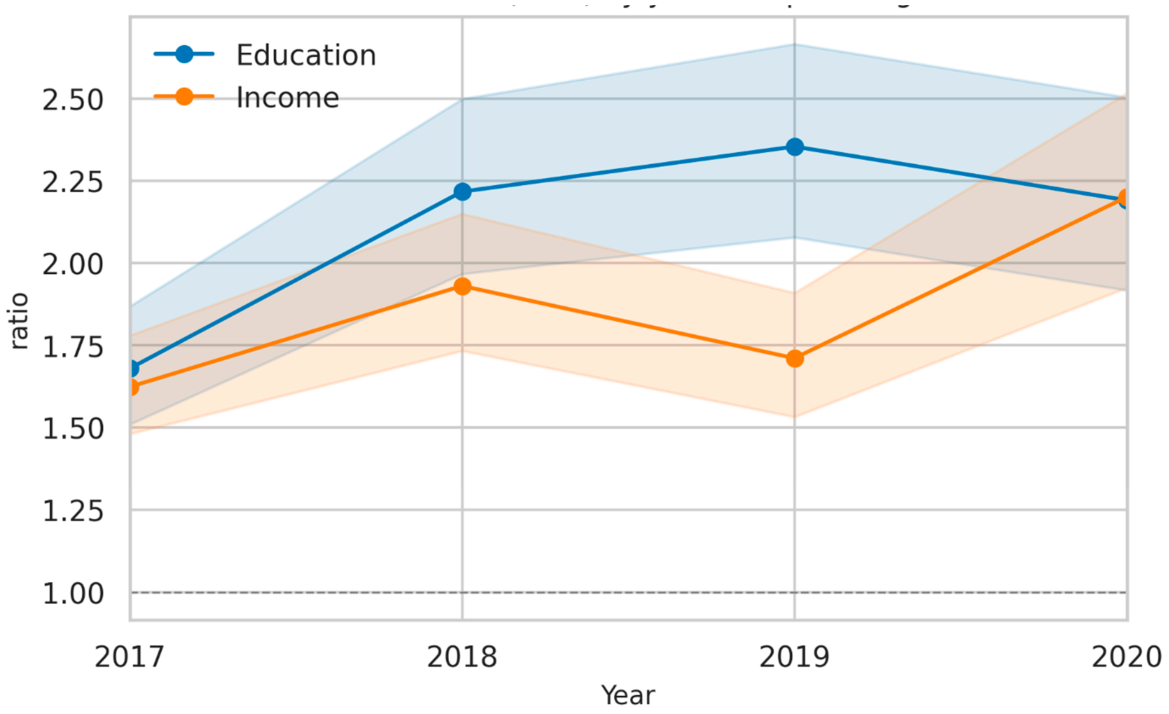

Figure S2. HC/HIT-05: Relative Index of Inequality by year, equal-category weights.

Note. RII values greater than 1 indicate higher estimates among more advantaged socioeconomic groups. The horizontal reference line indicates no relative socioeconomic gradient.

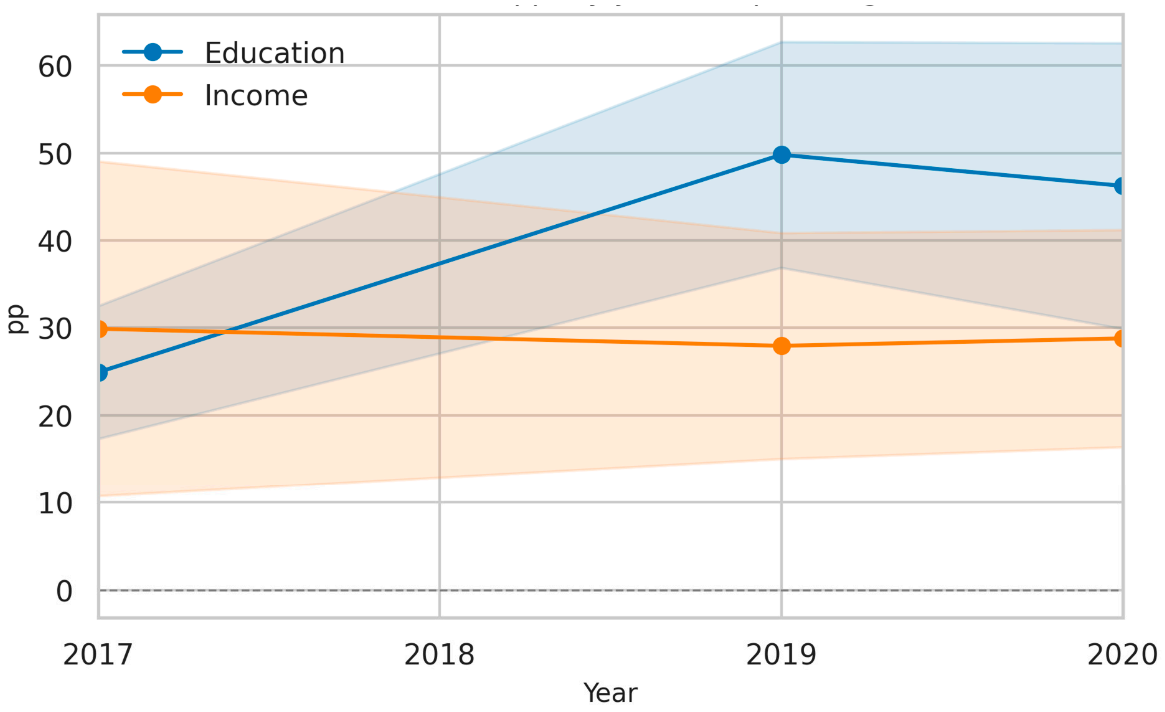

Figure S3. HC/HIT-06: Slope Index of Inequality by year, equal-category weights.

Note. SII values are expressed in percentage points. The horizontal reference line indicates no absolute socioeconomic gradient. Bands represent 95% confidence intervals where available.

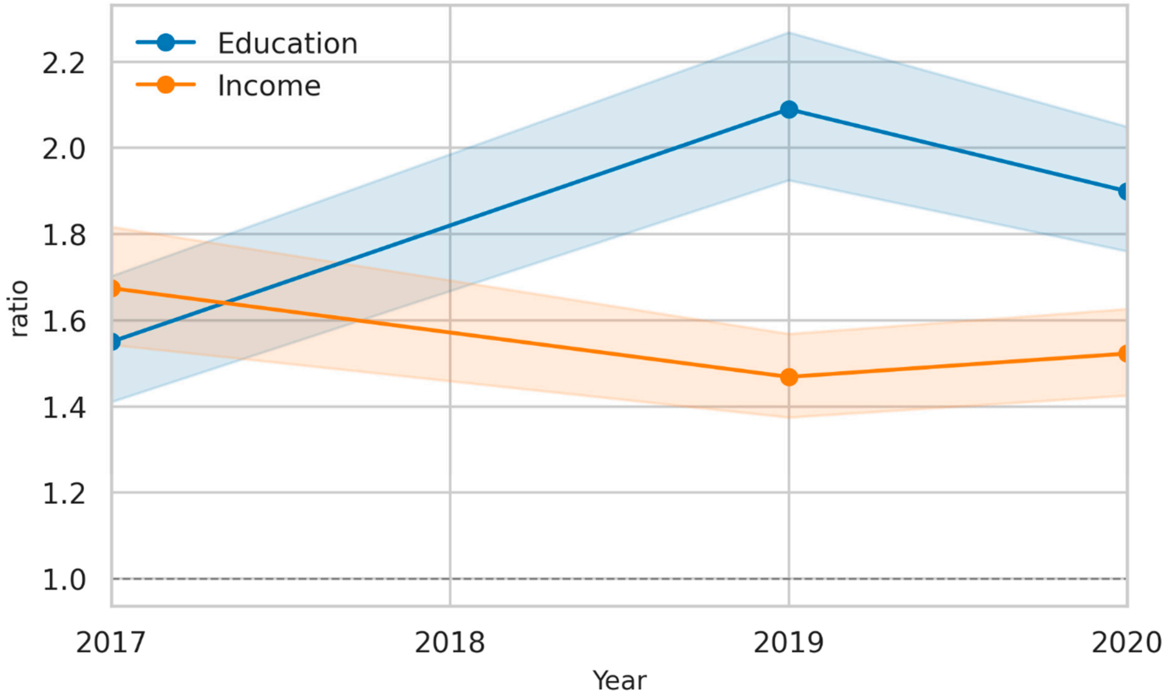

Figure S4. HC/HIT-06: Relative Index of Inequality by year, equal-category weights.

Note. RII values greater than 1 indicate higher estimates among more advantaged socioeconomic groups. The horizontal reference line indicates no relative socioeconomic gradient.

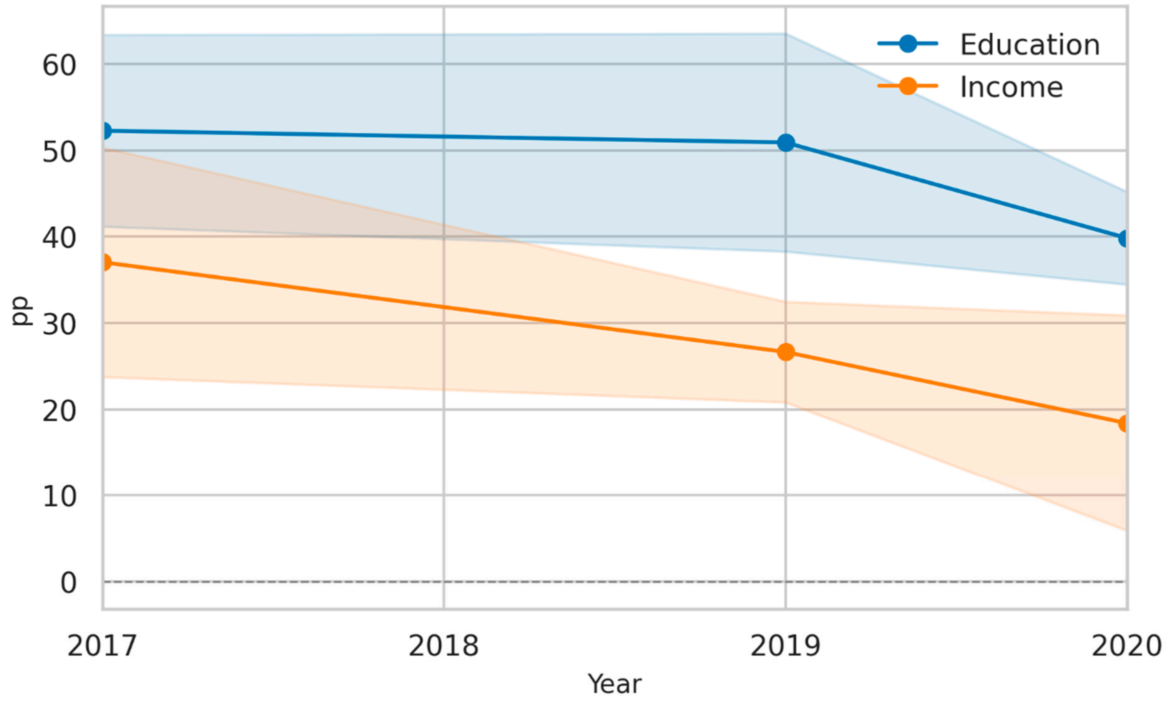

Figure S5. HC/HIT-07: Slope Index of Inequality by year, equal-category weights.

Note. SII values are expressed in percentage points. The horizontal reference line indicates no absolute socioeconomic gradient. Bands represent 95% confidence intervals where available.

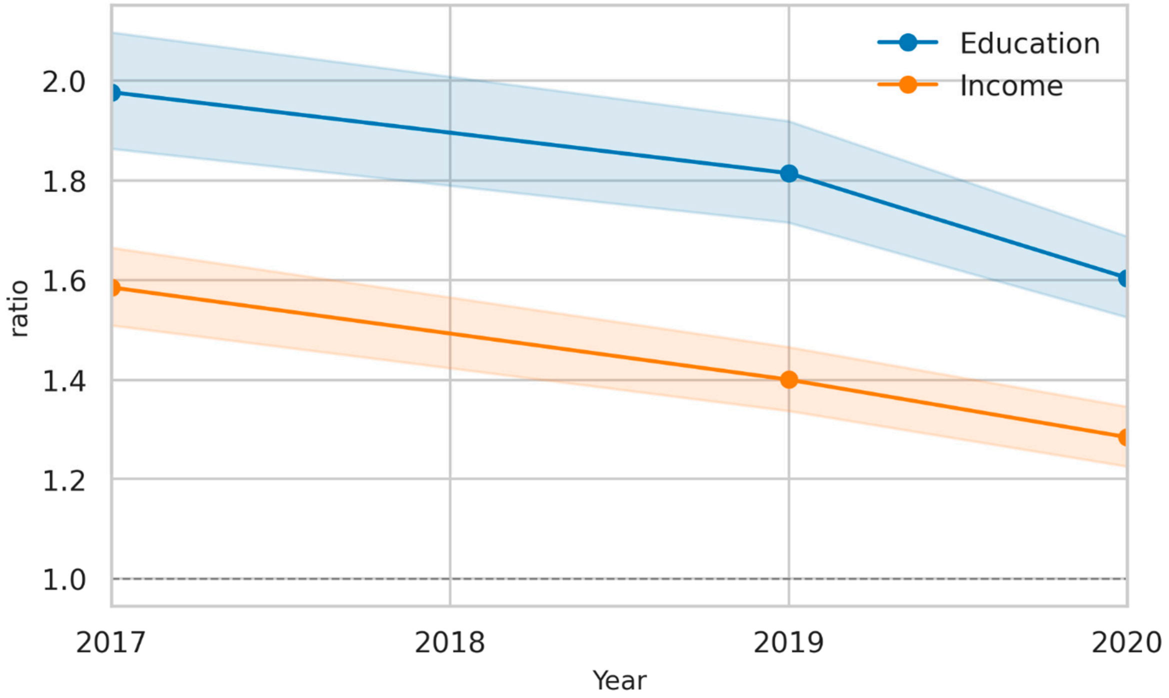

Figure S6. HC/HIT-07: Relative Index of Inequality by year, equal-category weights.

Note. RII values greater than 1 indicate higher estimates among more advantaged socioeconomic groups. The horizontal reference line indicates no relative socioeconomic gradient.

S7. Funnel plot results

Available 2020 funnel plots are provided in Supplementary Figures S7–S9. Selected sensitivity results are provided in Supplementary Tables S4 and S5.

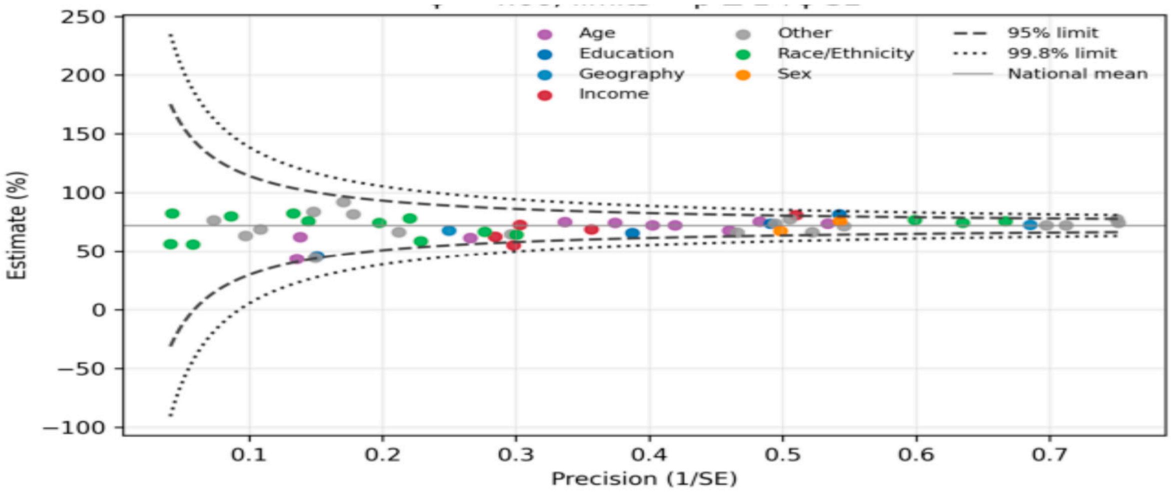

Figure S7. Funnel plot for HC/HIT-05, 2020.

Note. Points represent subgroup estimates. The horizontal line represents the national estimate. Curves represent overdispersion-adjusted control limits using the primary 10% Winsor specification. Points outside adjusted limits are interpreted as monitoring signals, not causal outliers.

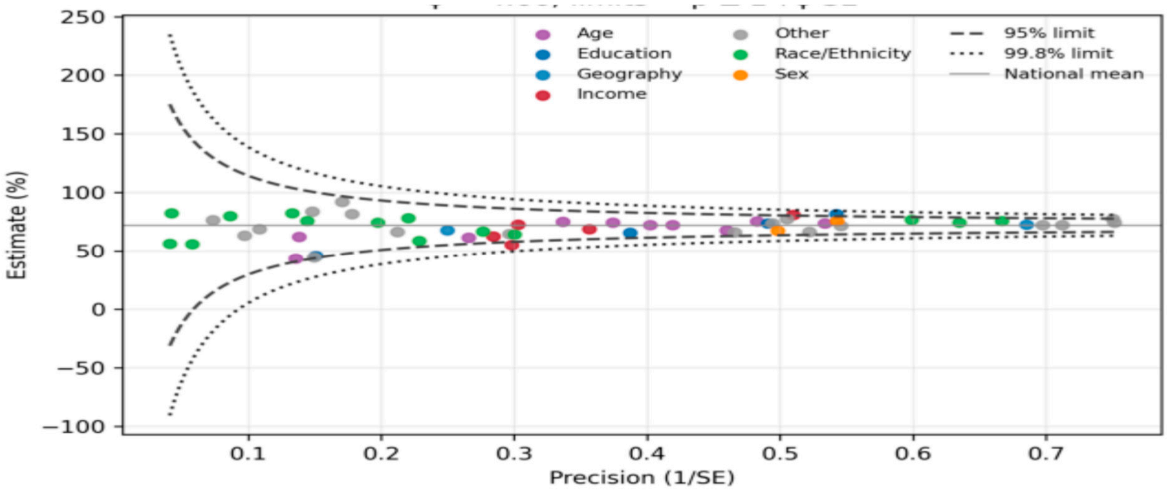

Figure S8. Funnel plot for HC/HIT-06, 2020.

Note. Points represent subgroup estimates. The horizontal line represents the national estimate. Curves represent overdispersion-adjusted control limits using the primary 10% Winsor specification. Points outside adjusted limits are interpreted as monitoring signals, not causal

outliers.

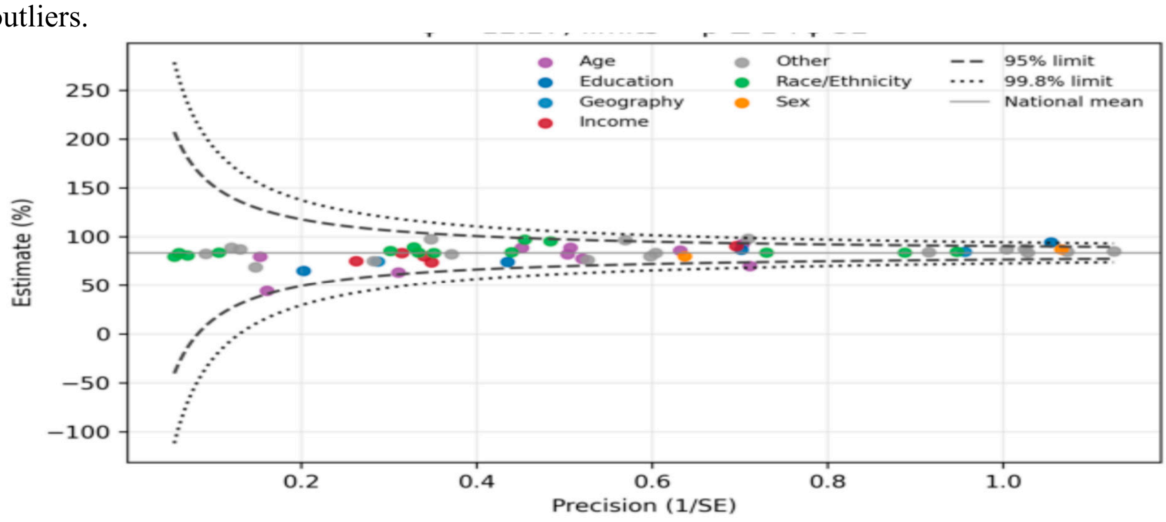

Figure S9. Funnel plot for HC/HIT-07, 2020.

Note. Points represent subgroup estimates. The horizontal line represents the national estimate. Curves represent overdispersion-adjusted control limits using the primary 10% Winsor specification. Points outside adjusted limits are interpreted as monitoring signals, not causal outliers.

S8. Sensitivity analyses

S8.1 Overdispersion Winsor specifications

The earlier Appendix C includes overdispersion estimates under 5%, 10%, and 20% Winsor specifications for selected indicator-years. These estimates support the conclusion that φ values exceeded 1 and that overdispersion adjustment was warranted.

Table S4. Selected overdispersion sensitivity estimates across Winsor specifications

Indicator	Year	Winsor 5%	Winsor 10%	Winsor 20%
**HC/HIT-05**	2017	1.42	1.35	1.28
**HC/HIT-05**	2018	1.38	1.32	1.25
**HC/HIT-06**	2017	1.45	1.40	1.33
**HC/HIT-07**	2017	1.50	1.44	1.36

Note. The 10% Winsor specification was used as the primary adjustment. Values above 1 indicate extra-binomial variability. These are selected sensitivity estimates available from the earlier analysis output.

S8.2 Funnel outlier counts

The earlier Appendix C also includes outlier counts beyond 95% and 99.8% limits under the 10% Winsor specification for selected indicator-years.

Table S5. Selected funnel outlier counts under the primary 10% Winsor specification

Indicator	Year	Winsor level	>95%	>99.8%
**HC/HIT-05**	2017	10%	14	6
**HC/HIT-05**	2018	10%	14	4
**HC/HIT-06**	2017	10%	18	8
**HC/HIT-07**	2017	10%	23	19

Note. Outlier counts are monitoring signals only. They should not be interpreted as causal effects or subgroup rankings. These are selected outlier-count outputs available from the earlier analysis.
